# Moderating Factors for the Effectiveness of Brief Psychological Intervention for People With Probable Personality Disorder: Secondary Analysis of Data From the SPS Trial

**DOI:** 10.1002/pmh.70096

**Published:** 2026-08-17

**Authors:** Ryan Williams, Kirsten Barnicot, Mark Sampson, Gill Attwood, Lisle Scott, Gordon Gunnarsen, Snehal P. Pandya, Verity Leeson, Mike J. Crawford

**Affiliations:** ^1^ Division of Psychiatry Imperial College London London UK; ^2^ Department of Health Service & Population Research King's College London UK; ^3^ Department of Population Health and Policy City St George's University of London London UK; ^4^ Community Enhanced Rehabilitation Team Mersey Care NHS Foundation Trust UK; ^5^ Complex Needs Service Oxford Health NHS Foundation Trust UK; ^6^ Coventry and Warwickshire Partnership NHS Trust UK; ^7^ Department of Psychology Brunel University of London UK

**Keywords:** brief intervention, early intervention, emotional dysregulation, personality disorder, psychological therapy, social functioning

## Abstract

Brief interventions have been proposed as a means of increasing access to psychological treatment for people with personality disorder. However, evidence of their effectiveness is limited to date. A recent large‐scale trial of Structured Psychological Support (SPS) for people with probable personality disorder showed little evidence of patient benefit. However, questions remain about whether shorter term psychological interventions may be suitable for some patients. We therefore conducted a secondary analysis of trial data to examine whether baseline level of personality dysfunction, duration of prior contact with mental health services and practitioner treatment sequence were associated with differential outcomes from SPS. Data from 336 participants recruited across seven NHS sites in England were analysed using multiple linear regression. We added interaction terms to examine moderation of treatment effects on social functioning and emotional dysregulation at 6 months. There was no clear evidence that practitioner treatment sequence influenced outcomes. There was also no strong statistical evidence that baseline personality dysfunction moderated treatment response, although exploratory analyses found weak evidence that SPS may be advantageous for emotional dysregulation among participants with lower levels of personality dysfunction. In addition, there was weak evidence that SPS was associated with improved social functioning among participants with shorter histories of contact with mental health services. Further research is needed to examine whether shorter term psychological interventions have a role for people with mild personality disorder or as part of early intervention pathways for people at an earlier stage of their contact with mental health services.

## 1 Introduction

Long‐term psychological treatments have been shown to benefit people with personality disorder (Cristea et al. 2017; Stoffers‐Winterling et al. 2022). However, such treatments are resource‐intensive and challenging to deliver, require sustained engagement over extended durations, and many people are unable to access or complete them (Barnicot et al. 2011; Evans et al. 2017; McMurran et al. 2010). This has generated interest in the development of briefer and more accessible interventions, and several short‐term approaches have been evaluated (Kivity et al. 2025; Kramer, Kolly, et al. 2022; Spong et al. 2021). While some studies suggest potential benefit, findings from larger pragmatic trials have been less supportive (Spong et al. 2021; Zhang et al. 2025). Questions therefore remain about whether brief interventions are effective in routine practice and who may be most likely to benefit from them.

Structured Psychological Support (SPS) was developed as a brief individual intervention drawing on psychological skills used in longer‐term evidence‐based treatments. During the sessions the practitioner develops a person‐centred formulation. They then work with the patient to agree a psychological focus for future meetings as well as providing information and advice about what steps they could take to improve their mental health. A feasibility trial of SPS found evidence of patient benefit (Crawford et al. 2020). In a subsequent multicentre randomised controlled trial conducted across seven NHS sites in England, 336 participants with probable personality disorder were allocated to SPS plus treatment‐as‐usual or enhanced treatment‐as‐usual (Crawford et al. 2024). No significant effect was found for SPS for the primary outcome of social functioning at 6 or 12 months, although modest short‐term improvements were observed in emotional dysregulation and self‐rated global improvement (Crawford et al. 2026). However, neutral findings in pragmatic trials may obscure clinically important heterogeneity, or benefits within specific subgroups.

Discussions within the research team and contributions from independent commentators highlighted factors that may have contributed to the negative findings of the trial. These included the severity and complexity of the participants who took part in the study (Luyten et al. 2026) and the stage at which participants were offered the intervention (Boone et al. 2025). Study participants had an average of over 13 years between their first contact with mental health services and taking part in the trial, and it has been argued that low‐intensity interventions such as SPS may be more effective if they are delivered during the earlier stages of an individual's mental health journey. Finally, most practitioners who delivered study treatments worked with only one or two participants. It was suggested that practitioners may not have been able to develop the familiarity and confidence in using SPS that would be needed to deliver benefits for patients (Crawford et al. 2026). Previous research suggests that therapist experience within trials may influence outcomes, with less favourable outcomes seen among early participants of trials compared to later participants (Gold et al. 2012).

We therefore conducted a secondary analysis of data from the SPS trial to examine whether baseline level of personality dysfunction, duration of prior service contact and practitioner treatment sequence were associated with differential outcomes following SPS.

## 2 Method

### 2.1 Study Design

This paper reports a secondary analysis of data collected in the SPS randomised controlled trial. Full details of the trial methods and findings have been reported elsewhere (Crawford et al. 2026; Crawford et al. 2024; Crawford et al. 2018). In the present study we examined whether selected participant‐ and practitioner‐level factors were associated with differential treatment outcomes following SPS.

The objective of the SPS trial was to evaluate the clinical effectiveness of a brief psychological intervention delivered in secondary mental health services for people with probable personality disorder. Following recruitment, participants were randomly allocated to one of two trial arms: treatment‐as‐usual along with a single crisis planning session (‘enhanced treatment‐as‐usual’, ETAU) or treatment‐as‐usual plus SPS.

Participants were interviewed at baseline, 6 and 12 months using standardised measures of social functioning, emotional dysregulation, quality of life and several mental health symptom scales. The trial found no evidence of an overall advantage for SPS on the primary outcome of social functioning, although modest short‐term improvements were observed on some secondary outcomes.

Participants were recruited from primary and secondary care mental health services delivered by seven state‐funded sites across England. Inclusion criteria included being aged 18 years or over and meeting parent trial eligibility criteria for probable personality disorder. Individuals were excluded from participating in the parent trial if they did not meet criteria for probable personality disorder using a score of four or more on the Standardised Assessment of Personality Disorder Abbreviated Scale (SAPAS) (Fok et al. 2015); had a co‐existing organic or psychotic mental disorder; were already receiving or about to receive psychological treatment for personality disorder (on a waiting list of less than one year); or were already taking part in another clinical trial or interventional research study. Participants were required to be able to provide informed consent and to participate in baseline assessment procedures. A total of 336 participants were randomised in the parent trial and formed the sample for the present secondary analyses, subject to availability of outcome and moderator data.

Participants randomised to SPS were offered up to 10 individual sessions delivered over a brief treatment period by trained staff working within participating services. SPS was developed as a structured, low‐intensity intervention drawing on psychological skills used in longer‐term evidence‐based treatments for personality disorder. Sessions focused on engagement, collaborative understanding of difficulties, crisis planning, emotional regulation and interpersonal coping strategies. Practitioners were provided with training and supervision in the model during the study. Enhanced treatment‐as‐usual comprised access to standard secondary mental health care together with a single crisis planning session. Usual care could include care coordination, psychiatric review, medication management and referral to other local interventions according to clinical need.

### 2.2 Measures and Moderators

The primary outcome for the main trial and for the present analyses was social functioning, measured using the Work and Social Adjustment Scale (WSAS). The WSAS is a five‐item self‐report measure of functional impairment across work, home management, social leisure, private leisure and close relationships, with higher scores indicating greater impairment (Mundt et al. 2002). The secondary outcome for this analysis was emotional dysregulation, measured using the Difficulties in Emotion Regulation Scale‐16 (DERS‐16). Higher scores on DERS‐16 indicate greater difficulties in emotional regulation (Bjureberg et al. 2016).

We focused on 6‐month outcomes rather than 12 months as this represented the earliest follow‐up after participants had the opportunity to complete the intervention and therefore provided the clearest estimate of initial treatment response (with 12‐month outcomes providing a more robust indicator of durability). Restricting moderation analyses to a single follow‐up reduced the number of statistical comparisons and simplified interpretation of exploratory moderator effects compared to multiple timepoints. This timepoint was prespecified in the Statistical Analysis Plan.

Potential moderators were selected a priori on clinical grounds and in response to hypotheses arising from publication of the main trial findings. Baseline level of personality dysfunction was examined using the SAPAS, with higher scores indicating greater severity (Fok et al. 2015). Duration of prior contact with secondary care mental health services was examined using the recorded number of years since first contact with services. To examine whether practitioner experience during the trial influenced outcomes, therapist allocation records were linked to trial data for participants allocated to SPS. Participants were classified according to whether they were the first, second, or third/subsequent participant treated by the practitioner delivering SPS.

### 2.3 Statistical Analysis

A statistical analysis plan for this secondary analysis was agreed prior to modelling. Participants were analysed according to their randomised allocation, irrespective of treatment uptake, consistent with the intention‐to‐treat principle. Separate linear regression models were fitted for each outcome: social functioning (WSAS) and emotional dysregulation (DERS‐16) at 6 months. In each model, the outcome score was regressed on treatment allocation while adjusting for baseline score on the corresponding measure, study site, age and gender, using an analysis of covariance approach. Potential moderation of treatment effect was examined by adding interaction terms between treatment allocation and each candidate moderator. For baseline level of personality dysfunction, an additional sensitivity analysis was completed restricting the sample to participants with SAPAS scores of 4–5, representing a group with ‘mild’ Personality Disorder (Olajide et al. 2018) for whom brief psychological interventions may be particularly appropriate within a stepped‐care model.

To examine the effects of practitioner experience, therapist allocation records were linked to trial data for participants randomised to SPS. Participants were classified according to whether they were the first, second, or third/subsequent participant treated by the practitioner delivering SPS. This variable was entered as a categorical exposure, generating a four‐level comparison: enhanced treatment‐as‐usual, SPS first case, SPS second case and SPS third/subsequent case.

In an additional exploratory analysis, outcomes among participants allocated to SPS were compared according to practitioner treatment sequence alone. These models excluded participants allocated to enhanced treatment‐as‐usual and examined whether outcomes differed between participants treated as a practitioner's first, second, or third/subsequent case after adjustment for baseline score, study site, age and gender. For continuous moderators, adjusted marginal estimates were generated at selected values of the moderator to aid interpretation of modelled treatment differences across the observed range of exposure. Regression coefficients with 95% confidence intervals and associated p values are reported. As these analyses were exploratory and secondary to the main trial outcomes, findings were interpreted cautiously, with emphasis placed on effect sizes, confidence intervals and consistency of patterns rather than statistical significance alone. All analyses were conducted using R version 4.6.0 (R Core Team 2024).

## 3 Results

A total of 336 participants were randomised in the parent trial, including 180 allocated to SPS and 156 allocated to ETAU. Six‐month outcome data were available for 286 participants (85.1%) for WSAS and 285 participants (84.8%) for DERS‐16. Among participants allocated to SPS, therapist‐order information was available for 179 participants (participants allocated to ETAU did not have a therapist). Of these, 77 were the first participant treated by that practitioner during the trial, 50 were the second, and 52 were the third or subsequent participant. As reported in the parent trial, baseline demographic and clinical characteristics of the two trial arms were broadly comparable. Mean age was 34.4 years in the SPS group and 35.1 years in the ETAU group. Mean duration of prior contact with mental health services was 14.0 and 12.8 years respectively (with a range of < 1 to 50 years), and mean SAPAS scores were similar across groups. A summary of baseline characteristics is given in Table 1—more detailed breakdowns are published elsewhere (Crawford et al. 2026). A summary of regression outputs for all moderator variables is given in Table 2.

**TABLE 1:** Baseline characteristics. ETAU = enhanced treatment‐as‐usual. SPS = Structured Psychological Support. SAPAS = Standardised Assessment of Personality—Abbreviated Scale. WSAS = Work and Social Adjustment Scale. DERS‐16 = Difficulties in Emotion Regulation Scale‐16.

	ETAU *n* = 156	SPS *n* = 180	Total *N* = 336
**Age, years**	35.1 (13.6)	34.4 (13.0)	34.8 (13.2)
**Gender**, n (%)			
Male	36 (23%)	39 (22%)	75 (22%)
Female	117 (75%)	134 (74%)	251 (75%)
Non‐binary or other	3 (2%)	7 (4%)	10 (3%)
**Ethnicity**, n (%)			
White	129 (83%)	152 (84%)	281 (84%)
Mixed	11 (7%)	8 (4%)	19 (6%)
Asian	9 (6%)	7 (4%)	16 (5%)
Black	6 (4%)	11 (6%)	17 (5%)
Other ethnic group	1 (1%)	2 (1%)	3 (1%)
**Employment status**, n (%)			
Not working or in education	80 (51%)	82 (46%)	162 (48%)
Employed, voluntary work, or education	76 (49%)	97 (54%)	173 (52%)
Missing	0	1 (1%)	1 (< 1%)
**Duration of contact with mental health services, years**	12.8 (9.6)	14.0 (10.9)	13.5 (10.4)
**SAPAS score – mean (SD)**	6.29 (1.20)	6.42 (1.13)	6.35 (1.16)
4	15 (9.6)	8 (4.4)	23 (6.8)
5	23 (14.7)	32 (17.8)	55 (16.4)
6	47 (30.1)	54 (30.0)	101 (30.1)
7	44 (28.2)	49 (27.2)	93 (27.7)
8	27 (17.3)	37 (20.6)	64 (19.0)
**WSAS score**	29.67 (6.84)	30.02 (6.01)	29.86 (6.40)
**DERS‐16 score**	65.13 (10.02)	65.72 (9.95)	65.45 (9.97)

**TABLE 2:** Regression outputs for moderators of 6‐month outcomes. Models adjusted for baseline outcome score, site, age and gender. In section A, the SPS main effect represents the fitted SPS‐versus‐ETAU difference if the moderator variable were set to zero, while the moderator main effect represents the association between the moderator and outcome within the ETAU group. Interaction estimates represent the additional change in the fitted SPS treatment effect associated with a one‐unit increase in the moderator variable. In section B, omnibus *p*‐values for practitioner‐order analyses are derived from overall tests of practitioner‐sequence category within adjusted regression models. Lower WSAS and DERS‐16 scores indicate better outcomes.

A. Participant‐level moderation analyses				
Moderator × outcome	SPS main effect β (95% CI)	Moderator main effect β (95% CI)	Interaction β (95% CI)	*p* value for interaction
Contact time × WSAS	−2.64 (−5.21 to −0.08)	0.11 (−0.02 to 0.25)	0.13 (−0.03 to 0.28)	0.106
Contact time × DERS‐16	−3.30 (−7.45 to 0.85)	0.11 (−0.11 to 0.32)	0.03 (−0.21 to 0.28)	0.794
SAPAS × WSAS	−5.50 (−16.03 to 5.02)	−0.63 (−2.27 to 1.01)	0.79 (−0.56 to 2.14)	0.250
SAPAS × DERS‐16	−15.30 (−31.50 to 0.90)	−0.11 (−2.64 to 2.42)	1.38 (−0.69 to 3.45)	0.190

B. Practitioner‐order analyses			
Outcome	Overall practitioner‐order effect *p*‐value	SPS practitioner sequence	Exploratory adjusted difference vs. ETAU β (95% CI)
WSAS	0.370	First participant	−0.75 (−2.77 to 1.27)
		Second participant	0.61 (−1.72 to 2.95)
		Third+ participant	−2.06 (−4.46 to 0.34)
DERS‐16	0.215	First participant	−4.23 (−7.40 to −1.06)
		Second participant	−1.10 (−4.80 to 2.60)
		Third+ participant	−2.03 (−5.86 to 1.79)

### 3.1 Baseline Level of Personality Dysfunction

**FIGURE 1:**
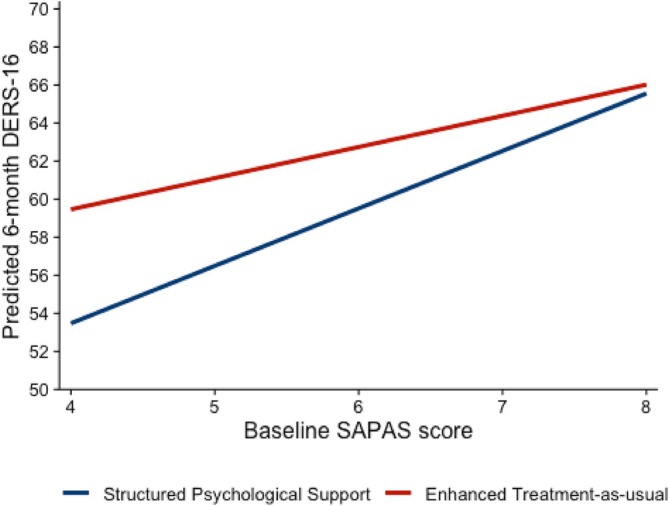
Modelled association between baseline SAPAS score and adjusted 6‐month DERS‐16 outcome according to treatment allocation. Predicted 6‐month emotional dysregulation scores (DERS‐16) derived from adjusted regression models examining interaction between treatment allocation and baseline SAPAS score. Higher scores indicate greater emotional dysregulation.

There was no strong statistical evidence that baseline SAPAS moderated the effect of SPS on social functioning or emotional dysregulation at 6 months. The treatment‐by‐SAPAS interaction coefficient for WSAS was 0.79 (95% CI −0.56 to 2.14, *p* = 0.250) and for DERS‐16 was 1.38 (95% CI −0.69 to 3.45, *p* = 0.190). However, in the sensitivity analysis excluding participants with baseline SAPAS > 5, there was weak evidence that treatment effects for emotional dysregulation varied according to baseline level of personality dysfunction, in the direction of diminishing benefit from SPS with increasing SAPAS score, with a coefficient of 1.63 (95% CI 0.02 to 3.96, *p* = 0.100) suggesting that any advantage associated with SPS may have been greater among participants with lower baseline SAPAS (illustrated graphically in Figure 1). Exploratory follow‐up estimates were consistent with this pattern. As baseline SAPAS score increased, the fitted difference between SPS and ETAU progressively narrowed. At a baseline SAPAS score of 4, the estimated difference in DERS‐16 between SPS and ETAU was −5.99 points in favour of SPS (95% CI −11.41 to −0.57; *p* = 0.031). At a SAPAS score of 5, the corresponding estimated difference was −4.61 points (95% CI −8.30 to −0.92; *p* = 0.015). At a SAPAS score of 6, the estimated difference was −3.23 points (95% CI −5.75 to −0.70; *p* = 0.013). At a SAPAS score of 7, the estimated difference was −1.84 points (95% CI −4.63 to 0.94; *p* = 0.193), and at a SAPAS score of 8, it was −0.46 points (95% CI −4.67 to 3.75; *p* = 0.829).

### 3.2 Duration of Prior Service Contact

**FIGURE 2:**
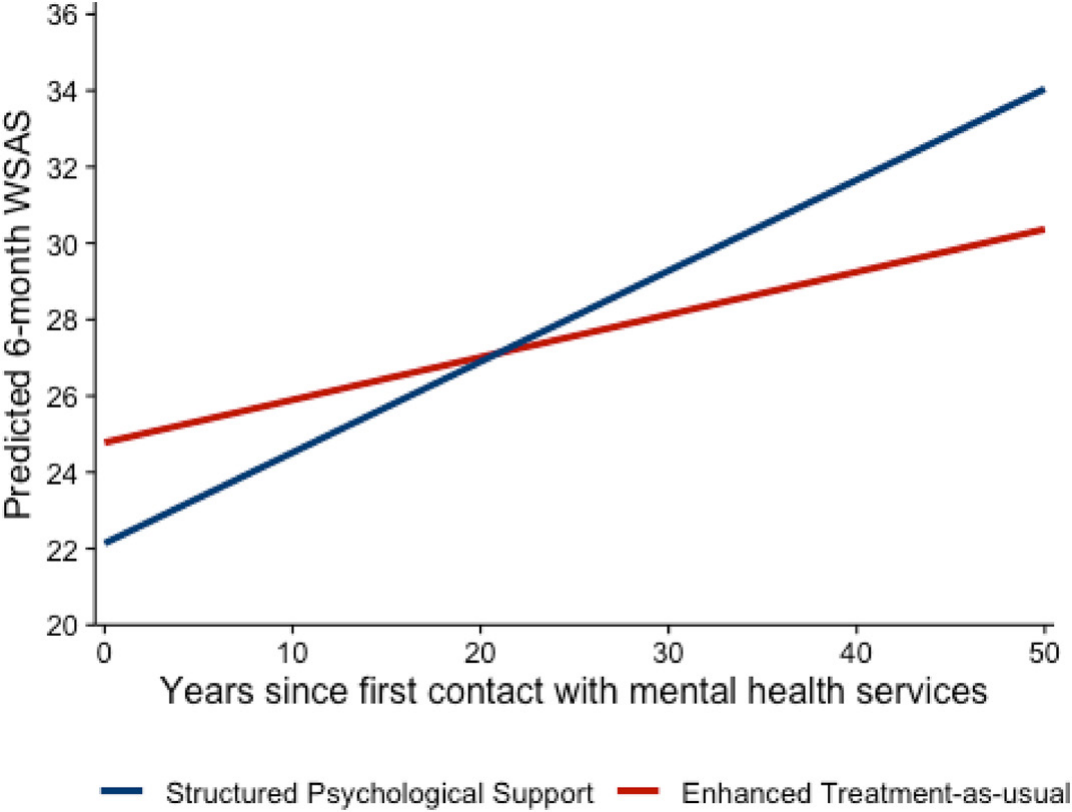
Modelled association between duration of prior mental health service contact and adjusted 6‐month WSAS outcome according to treatment allocation. Predicted 6‐month social functioning scores (WSAS) derived from adjusted regression models examining interaction between treatment allocation and years of prior contact with mental health services. Higher scores indicate poorer social functioning.

There was weak evidence that duration of prior contact with mental health services modified the effect of SPS on 6‐month social functioning. The treatment‐by‐contact‐time interaction coefficient was 0.13 per year (95% CI −0.03 to 0.28, *p* = 0.106), indicating that any benefit associated with SPS diminished with increasing duration of prior contact (illustrated graphically in Figure 2). Exploratory adjusted estimates supported this pattern. At five years or less since first service contact, the estimated WSAS difference between SPS and enhanced treatment‐as‐usual was −2.01 points in favour of SPS (95% CI −4.02 to 0.00; *p* = 0.050). At 10 years, the estimated difference was −1.38 points (95% CI −3.01 to 0.25; *p* = 0.098). At 20 years, the corresponding estimate was −0.12 points (95% CI −1.97 to 1.74; *p* = 0.903). At 30 years, the fitted estimate favoured ETAU (1.15 points, 95% CI −1.84 to 4.14; *p* = 0.450), with a similar pattern at 40 and 50 years, although estimates were imprecise due to relatively small numbers. There was no evidence that duration of prior service contact modified treatment effects on emotional dysregulation. The corresponding interaction coefficient for DERS‐16 was 0.03 (95% CI −0.21 to 0.28, *p* = 0.794).

### 3.3 Practitioner Treatment Sequence

Overall, no strong statistical evidence was found that practitioner treatment sequence was associated with outcome. Some isolated estimates approached or reached conventional levels of statistical significance (WSAS third/subsequent cases vs. ETAU; DERS‐16 first cases vs. ETAU). However, these findings were not suggestive of a consistent relationship between practitioner sequence and outcome. Overall F‐tests of the practitioner‐order factor within the adjusted regression models were non‐significant for both WSAS (*p* = 0.370) and DERS‐16 (*p* = 0.215). For social functioning, compared with enhanced treatment‐as‐usual, the adjusted difference in WSAS was −0.75 points (95% CI −2.77 to 1.27, *p* = 0.463) for participants treated as a practitioner's first SPS case, 0.61 points (95% CI −1.72 to 2.95, *p* = 0.607) for second cases and −2.06 points (95% CI −4.46 to 0.34, *p* = 0.092) for third or subsequent cases. For emotional dysregulation, compared with enhanced treatment‐as‐usual, adjusted differences in DERS‐16 were −4.23 points (95% CI −7.40 to −1.06, *p* = 0.009) for first cases, −1.10 points (95% CI −4.80 to 2.60, *p* = 0.560) for second cases and −2.03 points (95% CI −5.86 to 1.79, *p* = 0.296) for third or subsequent cases. In exploratory analyses restricted to participants allocated to SPS, practitioner treatment sequence was examined for social functioning only. No statistical evidence was found that treatment sequence was associated with outcome.

## 4 Discussion

In this secondary analysis of data from the SPS trial, we found limited evidence that outcomes following a brief psychological intervention varied according to participant characteristics and stage of service contact. In the parent trial, greater baseline personality dysfunction (as measured with SAPAS) was associated with poorer 6‐month outcomes irrespective of treatment allocation (Crawford et al. 2026). However, we found some suggestive (albeit weak) evidence that SPS was associated with greater improvement in emotional dysregulation among participants with lower baseline SAPAS scores, with this apparent advantage diminishing as baseline SAPAS increased. We also found weak evidence that SPS was associated with improved social functioning among participants with shorter histories of contact with mental health services.

These findings may help explain the largely negative overall results of the parent trial. Many participants in the SPS study had high levels of personality dysfunction, and many had already been in contact with services for prolonged periods. For individuals with more severe difficulties, sustained therapeutic engagement may be required before durable change can occur (Bateman and Fonagy 2000), and brief interventions may risk ’underdosing’ (Luyten et al. 2026).

However, we would also suggest that the findings should not necessarily be interpreted solely in terms of treatment ’dose’. It is possible that brief interventions and longer‐term therapies may act through partly distinct mechanisms and serve different clinical functions, with differential effects on emotional, interpersonal and functional domains across clinically distinct subgroups (Kramer, Eubanks, et al. 2022). For example, shorter interventions may be more effective in improving crisis management, whereas enduring changes in interpersonal and social functioning may require more prolonged contact to allow repeated opportunities for interpersonal learning and building therapeutic relationships (Kramer, Kolly, et al. 2022).

The findings are consistent with broader arguments for earlier intervention in personality disorder, involving identifying and treating personality difficulties before patterns of impairment become longstanding and more resistant to change (Chanen and McCutcheon 2013). Much of the evidence base has focused either on specialist longer‐term treatments or on crisis‐oriented service responses. Our results suggest that brief psychological interventions such as SPS may warrant further evaluation as part of a stepped care pathway for people presenting earlier (Grenyer et al. 2018) or with less severe personality disturbance, rather than being targeted at individuals with extensive prior service contact and high levels of complexity.

The novelty of the intervention and associated practitioner familiarity and confidence had also been considered as a possible explanation for the neutral findings of the parent trial. However, we found no clear evidence of a practitioner sequence effect (i.e., improved outcomes for those whose practitioner had already delivered SPS to other participants) to support this hypothesis.

These findings should be interpreted with appropriate restraint but may have some important implications. The results suggest that caution should be applied when considering brief interventions for people with moderate and severe personality disorder, especially those that have had significant prior contact with mental health services. However, they suggest that shorter term interventions may have a role within a broader service pathway, particularly for younger people presenting earlier or with milder forms of personality dysfunction. Such approaches may improve access to psychologically informed care, reduce delays in treatment and provide an acceptable first‐line option for some service users.

Further research is needed to test this proposition. Further trials of brief interventions should focus on populations at earlier stages of contact with services, include dimensional measures of severity and examine whether stepped‐care models can improve outcomes efficiently. Clarifying which patients are most likely to benefit from shorter term interventions remains an important priority for the development of accessible and effective services for people with personality disorder.

### 4.1 Strengths and Limitations

The study has several strengths. It draws on data from a large multicentre pragmatic randomised trial conducted in routine NHS settings, enhancing generalisability and clinical relevance. The analyses were theory‐informed and addressed plausible explanations for the main trial findings. We used standardised outcome measures and robust statistical methods including adjusting for key baseline covariates.

Limitations should also be acknowledged. These analyses were secondary and exploratory, and the trial was not explicitly powered to detect moderator effects. Some subgroup estimates were imprecise, particularly at the extremes of service‐contact duration and SAPAS score. SAPAS is a brief screening measure and may not fully capture dimensional severity of personality dysfunction. The secondary analysis restricted to participants with lower SAPAS scores involved a smaller subgroup and should therefore be interpreted cautiously. Duration of contact with mental health services is an imperfect proxy for stage of illness or chronicity and may additionally reflect broader structural and social determinants of service use. Finally, outcomes were examined at 6 months, and it is possible that different patterns of treatment moderation would be observed at longer‐term follow‐up. Future research could examine whether the moderators identified here are associated with the maintenance, as well as the initial emergence, of treatment effects.

### 4.2 Conclusions

These secondary analyses provide cautious support for the view that if brief psychological interventions are used, they may be best directed towards people with milder personality dysfunction and those earlier in their contact with mental health services. Although findings were exploratory, they suggest that shorter term treatments such as SPS may have a role within stepped care models as an accessible early intervention, rather than as an alternative to longer‐term treatment for people with more severe or longstanding difficulties. Further prospective research is needed to test this hypothesis.

## Author Contributions

M.C. designed and planned the study. R.W. analysed the data and wrote the first draft of the manuscript. All authors provided critical input and revisions to the draft manuscripts and approved the final manuscript. M.C. had final responsibility for the decision to submit for publication.

## Funding

This study was supported by a National Institute for Health Research Health Technology Assessment programme grant (reference NIHR133027).

## Ethics Statement

Ethical approval for the analysis necessary for this study was granted by the London Bromley Research Ethics Committee (IRAS ID 315951).

## Conflicts of Interest

The authors declare no conflicts of interest.

## Data Availability

Individual participant data and a data dictionary defining each field in the dataset will be made available upon reasonable request to the corresponding author.
